# Comparison of four rheological models for estimating viscosity and rheological parameters of microwave treated Basil seed gum

**DOI:** 10.1038/s41598-024-66690-x

**Published:** 2024-07-05

**Authors:** Fakhreddin Salehi, Maryam Tashakori, Kimia Samary

**Affiliations:** Department of Food Science and Technology, Faculty of Food Industry, Bu-Ali Sina University, Hamedan, Iran

**Keywords:** Basil seed gum, Bingham, Casson, Microwave, Power law, Chemical engineering, Computational biophysics, Carbohydrates

## Abstract

Dispersion of Basil seed gum has high viscosity and exhibits shear-thinning behavior. This study aimed to analyze the influence of microwave treatment (MT) at various time intervals (0, 1, 2, and 3 min) on the viscosity and rheological behavior of Basil seed gum dispersion (0.5%, w/v). The finding of this study revealed that the apparent viscosity of Basil seed gum dispersion (non-treated dispersion) reduced from 0.330 Pa.s to 0.068 Pa.s as the shear rate (SR) increased from 12.2 s^−1^ to 171.2 s^−1^. Additionally, the apparent viscosity of the Basil seed gum dispersion reduced from 0.173 Pa.s to 0.100 Pa.s as the MT time increased from 0 to 3 min (SR = 61 s^−1^). The rheological properties of gum dispersion were successfully modeled using Power law (PL), Bingham, Herschel–Bulkley (HB), and Casson models, and the PL model was the best one for describing the behavior of Basil seed gum dispersion. The PL model showed an excellent performance with the maximum r-value (mean r-value = 0.942) and the minimum sum of squared error (SSE) values (mean SSE value = 5.265) and root mean square error (RMSE) values (mean RMSE value = 0.624) for all gum dispersion. MT had a considerable effect on the changes in the consistency coefficient (k-value) and flow behavior index (n-value) of Basil seed gum dispersion (*p* < 0.05). The k-value of Basil seed gum dispersion decreased significantly from 3.149 Pa.s^n^ to 1.153 Pa.s^n^ (*p* < 0.05) with increasing MT time from 0 to 3 min. The n-value of Basil seed gum dispersion increased significantly from 0.25 to 0.42 (*p* < 0.05) as the MT time increased. The Bingham plastic viscosity of Basil seed gum dispersion increased significantly from 0.029 Pa.s to 0.039 Pa.s (*p* < 0.05) while the duration of MT increased. The Casson yield stress of Basil seed gum dispersion notably reduced from 5.010 Pa to 2.165 Pa (*p* < 0.05) with increasing MT time from 0 to 3 min.

## Introduction

Hydrocolloids, also called gums, are macromolecules that dissolve or disperse in water and form viscous dispersions or gels^1^. The uses of hydrocolloids in food systems is enormous, are very diverse, especially as thickeners, stabilizers, edible coatings, and fat replacers. Furthermore, hydrocolloids are used in a wide variety of applications due to their low cost and extensive functionality^2,3^. The annual consumption of hydrocolloids continues to grow; therefore, recent efforts have focused on finding new sources of more effective and economical hydrocolloids^1,4^. Basil seed gum is a hydrocolloid extracted from the seeds of *Ocimum basilicum* L. Aqueous dispersion of this gum produce high viscosity and shear-thinning behavior^4^.

Heat treatments, including microwave technology, are common in homes and are becoming more common in industrial applications^5,6^. Therefore, microwave irradiation reduces processing time, increases yield, and improves product quality than traditional processing methods^7^. Microwave treatment (MT) is now important for changing hydrocolloids because it can heat them evenly, is safe and easy to use, needs little maintenance, and is effective in changing the hydrocolloids' structure and improving their functional characteristics^8–11^. The effect of MT (700 W for 1–5 min) on flaxseed on the techno-functional characteristics of gum polysaccharides was studied by Yu, et al.^12^. Their findings revealed that when flaxseed was exposed to microwave for 1–5 min, the structure and properties of its polysaccharides changed, causing a significant impact on the surface appearance, molecular weight, and monosaccharide profiles of flaxseed gum. Also, when flaxseed was exposed to microwaves for 1–5 min, the rheological properties of flaxseed gum initially increased and then weakened. Yang, et al.^9^ studied how using a microwave changes the internal molecular structure of waxy maize starch and its physical characteristics. Their study found that the peak viscosity of the liquid decreased as the time that the starch was treated with microwaves expanded.

The rheological behavior of hydrocolloids is particularly important when they are used to modify textural properties, and it is also widely accepted that rheological properties play an important role in process design, evaluation and modeling and are considered a measure of product quality^1,4,13^. There are numerous investigations on the rheological properties of food hydrocolloids^1,2,4,13–15^. In our previous study^15^, we studied the effect of microwave pretreatment at different time intervals on the viscosity and rheological behavior of xanthan gum solution. The goal of this study was to explore the impacts of various microwave pretreatment intervals (0, 1, 2, and 3 min) on the viscosity and flow behavior of Basil seed gum dispersion.

## Materials and methods

### Preparation of gum dispersion

The collection of Basil seeds (also known as *Ocimum basilicum* L.) in this study was carried out in accordance with the law and formal approval of the Iranian National Standards Organization. These seeds were purchased from the market at Hamedan, Iran, and any dirt or other unwanted items were removed during the cleaning process. Then, the seeds were put in water for 20 min at a temperature of 25 °C, using 1 part of the seeds for every 20 parts of water. The Basil seed gum was taken out from the seeds by using a machine called an extractor (FJ-479, Tulips, Iran). This machine has a rotating disc that scrapes the gum off the surface of the seeds. The dispersion was dehydrated in an oven (Shimaz, Iran) with air blowing at 60 °C and then the gum powder was ground, packaged, and stored in a cool, dry place. The Basil seed gum powder was mixed with distilled water to make a dispersion (0.50%, w/v), using a stirrer.

### Microwave treatment (MT)

To use the microwave to treat the Basil seed gum, a microwave device (Gplus, Model; GMW-M425S.MIS00, Goldiran Industries Co., Iran) was employed. In this work, the impact of the MT time at four levels of 0, 1, 2, and 3 min, using a power of 440W, on the Basil seed gum dispersion was examined.

### Apparent viscosity

After each MT, the rheological properties of non-treated and microwave treated Basil seed gum dispersion were measured using a viscometer (Brookfield, DV2T, RV, USA) at 20 °C. The apparent viscosity and shear stress (SS) of Basil seed gum dispersion at various shear rates were measured using UL Adapter Kit^16^. All the measurements were performed over a wide range of shear rate from 12.2 to 171.2 s^−1^.

### Mathematical modeling

In this study, Power law (PL), Bingham, Herschel–Bulkley (HB), and Casson models (Table 1) were used to match the SS and shear rates (SR) results of the non-treated and microwave-treated Basil seed gum dispersion^3,4^. Rheological properties of Basil seed gum solutions were determined by applying nonlinear regression method based on minimizing sum of squared error (SSE) and root mean square error (RMSE) values in Matlab software (version R2012a). The experimental results were correlated for ease of use in rheological studies while maintaining appropriate accuracy using the function cftool (Curve Fitting Tool).

**Table 1:** Selected models for describing the rheological behavior of microwave-treated Basil seed gum.

Model name	Mathematical expression	Model parameters
Power law	$\tau = k\dot{\gamma }^{n}$	τ = Shear stress (Pa)
		k = Consistency coefficient (Pa.s^n^)
		γ̇ = Shear rate (s^−1^)
		n = Flow behavior index
Bingham	$\tau = \tau_{0B} + \eta_{B} \dot{\gamma }$	τ = Shear stress (Pa)
		τ_0B_ = Bingham yield stress (Pa)
		η_B_ = Bingham plastic viscosity (Pa.s)
		γ̇ = Shear rate (s^−1^)
Herschel–Bulkley	$\tau = \tau_{0H} + k_{H} \dot{\gamma }^{{n_{H} }}$	τ = Shear stress (Pa)
		τ_0H_ = Yield stress (Pa) k_H_ = Consistency coefficient (Pa.s^n^)
		γ̇ = Shear rate (s^−1^)
		n_H_ = Flow behavior index
Casson	$\tau^{0.5} = \tau_{0C}^{0.5} + \eta_{C} \dot{\gamma }^{0.5}$	τ = Shear stress (Pa)
		τ_0C_ = Casson yield stress (Pa)
		η_C_ = Casson plastic viscosity (Pa.s)
		γ̇ = Shear rate (s^−1^)

### Statistical analysis

Models parameters and errors were reported and Analysis of variance (ANOVA) was applied to acknowledge any significant difference among rheological parameters at *p* < 0.05. The SPSS (version 21) program was used for all statistical analysis. All experiments were replicated minimum three times. Differences between means were established using Duncan’s multiple range (*p* < 0.05).

## Results and discussion

### Apparent viscosity

**Figure 1:**
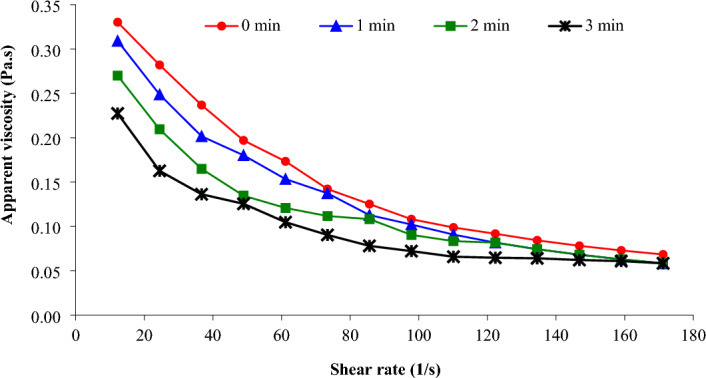
Impact of microwave pretreatment on the apparent viscosity of Basil seed gum solution.

Food thickening agents (gums) can demonstrate different behavior based on temperature, quantity, and state. The behavior of food thickening agents varies with temperature, quantity, and physical state^17,18^. Cui^19^ found the viscosity of the gum dispersion decreased with increasing the SR as the number of entangled chains reduced at high SRs. Figure 1 displays how the viscosity of Basil seed gum dispersion change when the shear is applied at different speeds. It can be seen that the apparent viscosity of Basil seed gum dispersion become less when it is stirred faster. The apparent viscosity reduced from 0.330 to 0.068 Pa.s with the SR increased from 12.2 to 171.2 s^−1^ (non-treated dispersion). Salehi and Inanloodoghouz^4^ studied the rheological properties of ultrasonic-treated aqueous dispersion of Basil seed gum. The finding of this study revealed that the apparent viscosity of aqueous dispersion of Basil seed gum reduced with increasing SR, indicating the shear-thinning behavior of this aqueous dispersion.

The selection of the appropriate hydrocolloid for a system depends not only on cost and safety, but also on the function and desired properties of the hydrocolloid in food products^1^. The influence of MT on the apparent viscosity of Basil seed gum dispersion is shown in Fig. 1. The MT of Basil seed gum dispersion reduces its viscosity. This behavior was observed under all conditions and after 3 min of pretreatment, resulting in a significant decrease in gum viscosity. The results show that when the MT time is increased from 0 to 3 min, the average apparent viscosity of the Basil seed gum dispersion reduced from 0.173 to 0.100 Pa.s (SR = 61 s^−1^). MT reduces the viscosity, which is likely due to molecular rearrangement limited to a portion of the hydrocolloid molecules^10^. The effect of MT on acid hydrolysis of faba bean starch was examined by González-Mendoza, et al.^20^. The finding of this study revealed that the lowest viscosity values for starch were achieved when combining more severe hydrochloric acid and microwave energy conditions.

### Mathematical modeling

Rheological data is necessary for calculations in all processes where liquid flow occurs (pump sizing, extraction, filtration, extrusion, purification, etc.) and plays an important role in the analysis of flow conditions in food processes such as pasteurization, evaporation and drying^1,13^.

**Figure 2:**
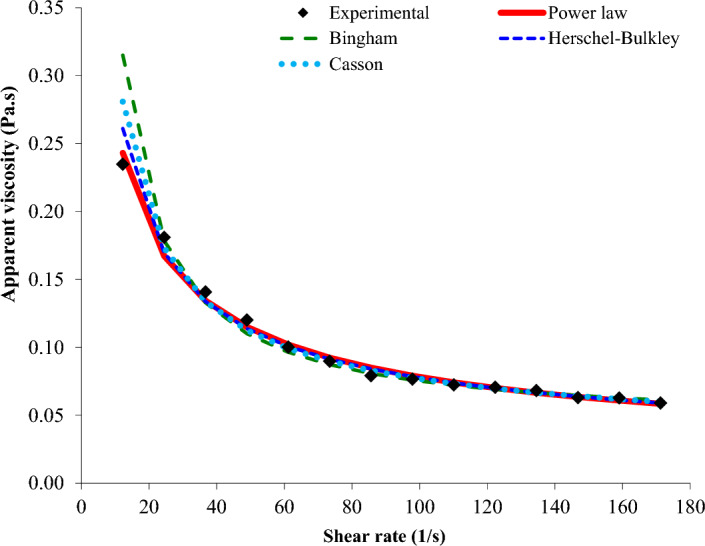
Fitting ability of various rheological equations to experimental data.

The flow behavior of Basil seed gum dispersion was effectively modeled using the PL, Bingham, HB, and Casson models, and the PL model was found as the better model to describe the flow behavior of Basil seed gum dispersion. Figure 2 shows the fit of rheological equations to the actual data. This figure shows that both the PL and HB equations were equally suitable in predicting the relationships between SS and SR data of microwave-treated Basil seed gum dispersion. Salehi, et al.^15^ reported that the HB model with the maximum r-value (higher than 0.9032) and the minimum SSE (lower than 0.7165) and RMSE (lower than 0.2552) has acceptable performance in modeling the flow behavior of microwave-treated xanthan gum solutions.

### Power law (PL) model

The PL model showed an excellent performance with the highest r-value (higher than 0.862) and the lowest SSE values (lower than 10.550) and RMSE values (lower than 0.938) for all gum dispersion (Table 2). Treatment with microwave had a significant effect on the change of consistency coefficient (k-value) and flow behavior index (n-value) of Basil seed gum dispersion (*p* < 0.05).

**Table 2:** Values of statistical parameters of rheological models for estimating shear stress data.

Rheological model	Microwave treatment time (min)	Sum of squared error (SSE)	Correlation coefficient (r)	Root mean square error (RMSE)
Power law	0	9.5063	0.8943	0.8890
	1	6.5987	0.9289	0.7315
	2	3.5290	0.9632	0.5338
	3	1.4254	0.9860	0.3398
Bingham	0	19.9367	0.7648	1.2887
	1	14.5833	0.8569	1.0976
	2	10.0843	0.8910	0.9108
	3	2.8623	0.9711	0.4811
Herschel–Bulkley	0	10.8163	0.8792	0.9905
	1	9.1517	0.8990	0.8641
	2	8.4987	0.9075	0.7738
	3	1.6716	0.9830	0.3786
Casson	0	14.6900	0.8322	1.1060
	1	10.3930	0.8866	0.9218
	2	6.4917	0.9312	0.7284
	3	1.7589	0.9822	0.3736

**Figure 3:**
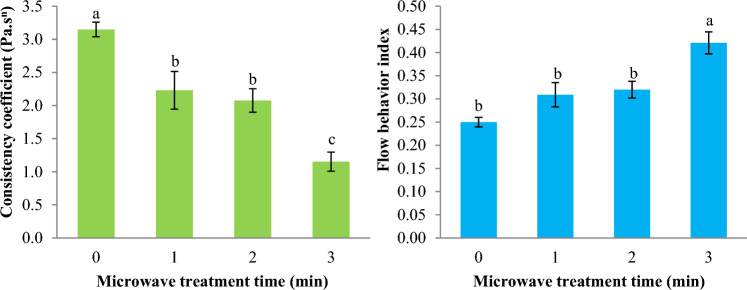
Impact of microwave treatment on the consistency coefficient (k) and flow behavior index (n) of Basil seeds gum solution (Power law model). Data are mean ± SD. Different letters indicate significant differences between microwave treatments at *p* < 0.05.

The impact of MT on the k-value of Basil seed gum dispersion is reported in Fig. 3. The k-value of Basil seed gum dispersion considerably decreased from 3.149 Pa.s^n^ to 1.153 Pa.s^n^ (*p* < 0.05) with increasing MT time from 0 to 3 min.

The PL equation shows that a fluid with shear-thinning behavior has a value of n less than 1^21^. The impact of MT on the n-value of Basil seed gum dispersion is reported in Fig. 3. The n-value of Basil seed gum dispersion increased significantly from 0.250 to 0.421 (*p* < 0.05) (decreases in shear-thinning behavior) while the duration of MT increased. The alteration within the k-value and n-value of the Basil seed gum dispersion may be due to the structural change of the gum during MT. Microwave energy is known to induce a series of physico-chemical reactions that lead to changes in the functional properties of gums in liquid food systems.

### Bingham model

The experimental values of SS versus SR for non-treated and treated Basil seed gum dispersion were fitted to the Bingham model and the constant coefficients of this equation were calculated. The mean values ​​of SSE, r, and RMSE for Basil seed gum dispersion were between 1.680 and 20.350, 0.710 and 0.986, and 0.374 and 1.302, respectively Table 2.

**Figure 4:**
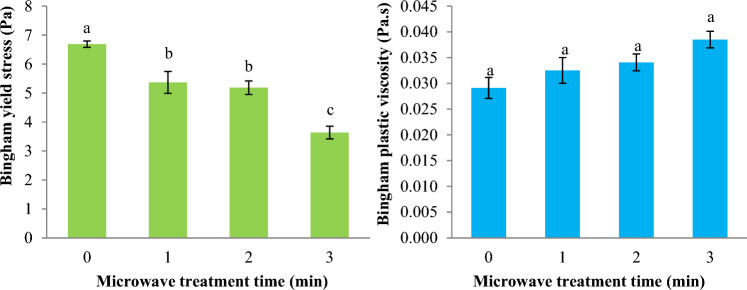
Impact of microwave treatment on the Bingham yield stress (τ_0B_) and Bingham plastic viscosity (η_B_) parameters of Basil seeds gum solution (Bingham model). Data are mean ± SD. Different letters indicate significant differences between microwave treatments at *p* < 0.05.

The impact of MT on the Bingham yield stress parameter (τ_0B_) of Basil seed gum dispersion is reported in Fig. 4. The Bingham yield stress parameters of Basil seed gum dispersion considerably decreased from 6.687 Pa to 3.636 Pa (*p* < 0.05) with increasing MT time from 0 to 3 min. In addition, the impact of MT on the Bingham plastic viscosity (η_B_) of Basil seed gum dispersion is reported in Fig. 4. The Bingham plastic viscosity of Basil seed gum dispersion increased significantly from 0.029 Pa.s to 0.039 Pa.s (*p* < 0.05) while the duration of MT increased.

### Herschel–Bulkley (HB) model

**Figure 5:**
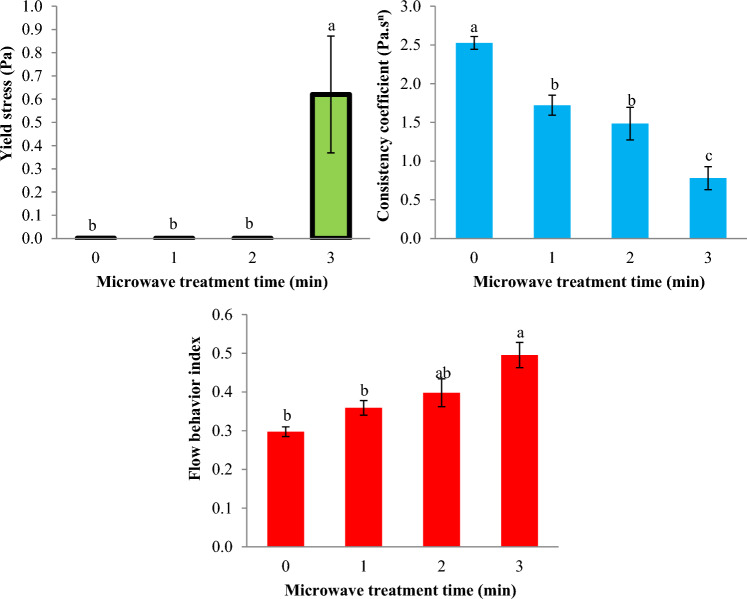
Impact of microwave treatment on the yield stress (τ_0H_), consistency coefficient (k_H_), and flow behavior index (n_H_) parameters of Basil seeds gum solution (Herschel–Bulkley model). Data are mean ± SD. Different letters indicate significant differences between microwave treatments at *p* < 0.05.

Based on the HB model, all Basil seed gum dispersion demonstrated shear-thinning behavior, described by the n-value (n_H_) lower than 0.573 (Fig. 5). The results of HB model showed that the values of the yield stress were between 0.001 Pa and 0.620 Pa. Mean values ​​of SSE, r, and RMSE for Basil seed gum dispersion ranged from 0.717–20.100, 0.761–0.994, and 0.255–1.352, respectively Table 2.

The impact of MT on the k-value of Basil seed gum dispersion is reported in Fig. 5. The k-value of Basil seed gum dispersion significantly reduced from 2.527 Pa.s^n^ to 0.780 Pa.s^n^ (*p* < 0.05) with increasing MT time from 0 to 3 min. In addition, the impact of MT on the n-value of Basil seed gum dispersion is reported in Fig. 5. The n-value of Basil seed gum dispersion increased significantly from 0.297 to 0.495 (*p* < 0.05) (decreases in shear-thinning behavior) while the duration of MT increased.

### Casson model

The rheological characteristics of hydrocolloids are extremely important because of the structural and textural properties of food products ^14^. The experimental values of SS versus SR for non-treated and treated Basil seed gum dispersion were fitted to the Casson model and the constant coefficients of this equation were calculated. Mean values ​​of SSE, r, and RMSE for Basil seed gum dispersion were between 0.813 and 15.540, 0.788 and 0.993, and 0.260 and 1.138, respectively (Table 2).

**Figure 6:**
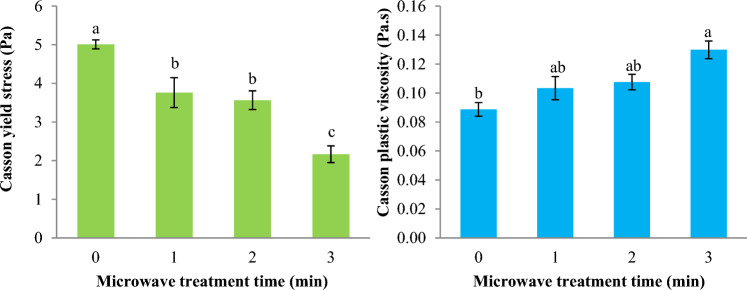
Impact of microwave treatment on the Casson yield stress (τ_0C_) and Casson plastic viscosity (η_C_) parameters of Basil seeds gum solution (Casson model). Data are mean ± SD. Different letters indicate significant differences between microwave treatments at *p* < 0.05.

The impact of MT on the Casson yield stress (τ_0C_) of Basil seed gum dispersion is reported in Fig. 6. The Casson yield stress of Basil seed gum dispersion notably reduced from 5.010 Pa to 2.165 Pa (*p* < 0.05) with increasing MT time from 0 to 3 min. In addition, the impact of MT on the Casson plastic viscosity (η_C_) of Basil seed gum dispersion is reported in Fig. 6. The Casson plastic viscosity of Basil seed gum dispersion increased significantly from 0.089 Pa.s to 0.130 Pa.s (*p* < 0.05) while the duration of MT increased. The effect of microwave pretreatment on the rheological behavior of xanthan gum solutions was investigated by Salehi, et al.^15^. The results showed that the Casson yield stress of xanthan gum solutions was between 1.378 Pa and 1.678 Pa, and the Casson plastic viscosity was between 0.048 Pa.s and 0.105 Pa.s.

## Conclusion

In the current work, the impact of MT on the rheological behavior of Basil seed gum dispersion was investigated. Basil seed gum dispersion showed the shear-thinning flow behavior. The utilization of microwave to the Basil seed gum dispersion reduces its viscosity. The finding of this study revealed that the PL model became the most accurate model to show the rheological behavior of Basil seed gum dispersion compared to three other confirmed rheological models with RMSE values between 0.259 and 0.938. The k-value values (PL and HB models) of the samples decreased significantly when the MT time was increased to 3 min (*p* < 0.05). The highest n-value (PL and HB models) was for the gum dispersion treated in the microwave for 3 min, and the lowest n-value was for the non-treated sample. Also, the Casson plastic viscosity of Basil seed gum dispersion increased significantly while the duration of MT increased.

## Data Availability

All data generated or analyzed during this research are included in this published manuscript.
